# Long non-coding RNA SNHG6 regulates the sensitivity of prostate cancer cells to paclitaxel by sponging miR-186

**DOI:** 10.1186/s12935-020-01462-x

**Published:** 2020-08-07

**Authors:** Chunhui Cao, Guanghai Sun, Chunlin Liu

**Affiliations:** Department of Urology, The Second People’s Hospital of Taizhou, No. 27, Jiankang Road, Jiangyan District, Taizhou, 225500 Jiangsu China

**Keywords:** Prostate cancer, SNHG6, MiR-186, PTX

## Abstract

**Background:**

Chemo-resistance is one of the main obstacles in the treatment of prostate cancer (PCa). Long non-coding RNA small nucleolar RNA host gene 6 (SNHG6) is involved in the chemo-resistance of various tumors. We aim to survey the role and underlying molecular mechanism of SNHG6 in PCa resistance to paclitaxel (PTX).

**Methods:**

The expression of SNHG6 and miR-186 was detected using quantitative real time polymerase chain reaction (qRT-PCR). The proliferation, migration, invasion, and apoptosis of PTX-resistant PCa cells were determined via 3-(4,5-dimethyl-2-thiazolyl)-2,5-diphenyl-2-H-tetrazolium bromide (MTT), transwell assay, or flow cytometry assay. Protein levels of CyclinD1, matrix metalloproteinase 9 (MMP9), Vimentin, E-cadherin, Cleaved-caspase-3 (Cleaved-casp-3) Cleaved-caspase-9 (Cleaved-casp-9), Multidrug Resistance associated Protein 1 (MRP1), and multidrug resistance-1 (MDR1) were assessed by western blot analysis. The relationship between SNHG6 and miR-186 were confirmed by dual-luciferase reporter assay. The role of SNHG6 in vivo was confirmed by xenograft tumor model.

**Results:**

SNHG6 expression was increased and miR-186 expression was reduced in drug-resistant PCa tissues and cells. SNHG6 knockdown elevated PTX-resistant PCa cells sensitivity to PTX in vitro and in vivo, and repressed proliferation, migration, and invasion of PTX-resistant PCa cells in vitro. Importantly, SNHG6 acted as a sponge of miR-186. Furthermore, miR-186 downregulation reversed SNHG6 silencing-mediated cell sensitivity to PTX, proliferation, migration, and invasion in PTX-resistant PCa cells.

**Conclusions:**

SNHG6 knockdown elevated the sensitivity of PTX-resistant PCa cells to PTX by sponging miR-186, indicating that SNHG6 might be a therapeutic target for PCa.

## Background

Prostate cancer (PCa) is the common male malignant tumor, with an estimated 1.3 million new cases and 359,000 deaths worldwide in 2018 [1]. Currently, PCa can be treated by radiotherapy, chemotherapy, surgical, and a combination of these methods. However, about 25% of PCa patients relapse within 5 years, and the recurrence of PCa and progression of castration-resistant are the main causes of PCa death [2, 3]. PTX is the first-line treatment drug for PCa. Moreover, the main cause of recurrence in PCa patients is resistance to PTX [4]. Consequently, exploring the underlying molecular mechanism of PCa chemo-resistance is of great vital to elevate the sensitivity of PCa patients to PTX.

Long non coding RNAs (lncRNAs), a class of transcripts longer than 200 nucleotides, cannot be translated into proteins. They mainly regulate mammalian protein expression through affecting protein-coding transcripts, protein transcription, pre-mRNA splicing, mRNA decay, and protein translation and degradation [5]. LncRNAs plays vital roles in human physiology and disease processes [6]. A substantial amount of evidence shows that lncRNAs are related to the chemo-resistance of diverse cancers, such as ovarian cancer [7], gastric cancer [8] cervical cancer [9], and hepatocellular cancer [10]. LncRNA small nucleolar RNA host gene 6 (SNHG6) has been reported to be abnormally expressed in colorectal cancer [11], osteosarcoma [12], and breast cancer [13]. Moreover, SNHG6 accelerated the progression of colorectal cancer by activating the TGF-beta/Smad pathway [14]. A recent study showed that SNHG6 facilitated the resistance of colorectal cancer cells to 5-fluorouracil [15]. However, the molecular mechanism of SNHG6 in the chemo-resistance of PCa to PTX has rarely been reported.

MicroRNAs (miRNAs), a group of short non-coding RNAs with a length of 19-25 nucleotides, mainly bind to the 3′-untranslated regions (UTR) of the target genes to regulate gene expression [16]. Increasing evidence has shown that some miRNAs play crucial roles in tumorigenesis processes [17]. MiRNAs is considered as potential diagnostic biomarkers and therapeutic targets for some diseases [18–20]. It was reported that miR-186 was associated with the chemo-resistance of a range of cancers [21–24]. Nevertheless, it is unclear whether miR-186 is involved in the chemo-resistance of PCa mediated by SNHG6.

Hence, the expression patterns of SNHG6 and miR-186 in drug-sensitivity tissues and drug-resistant tissues were explored. Moreover, the influence of SNHG6 on sensitivity of PTX-resistant PCa cells to PTX was probed. Furthermore, the mechanism of SNHG6/miR-186 axis in PCa cells was investigated.

## Materials and methods

### Patient PCa specimens

63 PCa tissue specimens used in this study were obtained from the Second People’s Hospital of Taizhou. The fresh specimens of PCa were verified by pathologist and stored at − 80 °C for further study. Whole blood (20 mL) was drawn from each PCa patients. To acquire serum, the blood sample was maintained at room temperature for 2 h and then centrifuged at 1200×*g* for 20 min. All PCa patients had signed informed consents. This study was approved by the research Ethics Committee of the Second People’s Hospital of Taizhou. According to the Response Evaluation Criteria in Solid Tumors (RECIST), PCa patients with PTX treatment were divided into 2 groups: 30 drug-sensitivity patients and 33 drug-resistant patients.

### Cell culture and treatment

Human PCa cell lines (PC-3 and DU145) were purchased from the American Type Culture Collection (Rockville, MD, USA). Roswell Park Memorial Institute (RPMI) 1640 medium (Thermo Fisher Scientific, Waltham, MA, USA) with 10% fetal bovine serum (FBS, Gibco, NY, USA) and 100 U/mL penicillin/streptomycin (Corning, NY, USA) was used for cell growth. The above cells were maintained in a humidified incubator with 5% CO_2_ at 37 °C. For PTX-resistant PCa cells (PC-3/R and DU145/R), it was produced by parental PC-3 or DU145 cells by gradually elevating the PTX concentration in the medium up to 30 nM, and PC-3/R and DU145/R cells were maintained in 5 nM PTX.

### Cell transfection

MiR-186 mimic (miR-186) and negative control mimic (miR-NC), as well as miR-186 inhibitor (anti-miR-186) and negative control inhibitor (anti-NC), were purchased from GenePharma (Shanghai, China). Small interference RNA (si-RNA) targeting SNHG6 (si-SNHG6), short hairpin RNA (sh-RNA) targeting SNHG6 (sh-SNHG6), and their corresponding negative control (si-NC and sh-NC) were obtained from GenePharma. Oligonucleotides were transfected into PC-3/R and DU145/R cells using lipofectamine 2000 reagent (Invitrogen, Carlsbad, CA, USA) based on the instruction of the manufacturer. The sequences were displayed as the following: si-SNHG6 (5′-CGCGAAGAGCCGTTAGTCATGCCGGTGTG-3′), si-NC (5′-UUCUCCGAACGUGUCACGUTT-3′), sh-SNHG6 (5′-CTGCGAGGTGCAAGAAAGCCT-3′), and sh-NC (5′-GTTCTCCGAACGTGTCACGTC-3′).

### Quantitative real-time polymerase chain reaction (qRT-PCR)

A total RNA of PCa specimens and cell lines was extracted using TRIzol reagent (Thermo Fisher Scientific) as following the manufacturer’s instructions. Pirmer-Script one step RT-PCR kit (Takara, Dalian, China) or MicroRNA Reverse Transcription Kits (Thermo Fisher Scientific) were applied to synthesize the first strand of complementary DNA. Next, qRT-PCR was executed with the Platinum SYBR Green qPCR SuperMix UDG (Invitrogen) in a Fast Real-time PCR 7300 System (Applied Biosystems, Foster City, CA, USA). The primers sequences used were listed as below: SNHG6: (F: 5′-CCTACTGACAACATCGACGTTGAAG-3′ and R: 5′-GGAGAAAACGCTTAGCCATACAG-3′); miR-186: (F: 5′-CCCGATAAAGCTAGATAACC-3′ and R: 5′-CAGTGCGTGTCGTGGAGT-3′); glyceraldehyde-3-phosphate dehydrogenase (GAPDH): (F: 5′-GACTCCACTCACGGCAAATTCA-3′ and R: 5′-TCGCTCCTGGAAGATGGTGAT-3′); U6 small nuclear RNA (snRNA) (F: 5′-GCTCGCTTCGGCAGCACA-3′, R: 5′-GAGGTATTCGCACCAGAGGA-3′). GAPDH or U6 was viewed as an internal control for SNHG6 and miR-186. Relative expression of SNHG6 and miR-186 was calculated by the 2^−ΔΔCt^ method.

### Western blot assay

Total protein of resistant PCa cell lines was extracted with RIPA lysis buffer (Beyotime, Shanghai, China). Total protein was separated by 10% sodium dodecyl sulphate–polyacrylamide gel electrophoresis (SDS-PAGE) and then transferred onto the polyvinylidene fluoride (PVDF) membranes (Millipore, Billerica, MA, USA). Then, the PVDF membranes were blocked in tris buffered saline tween (TBST) buffer with 5% non-fat dry milk for 1 h. Afterward, the membranes were incubated with primary antibodies at 4 °C overnight. After washing with TBST, the PVDF membranes were incubated with secondary antibodies: goat anti-rabbit IgG-HRP (ab6721, 1:10,000, Abcam, MA, USA) or goat anti-mouse IgG-HRP (ab205719, 1:5000, Abcam) for 1 h at 37 °C. The blots were determined using the electrochemiluminescent detection system (Thermo fisher scientific). The primary antibodies used were listed as below: anti-CyclinD1 (ab16663, 1:200, Abcam), anti-matrix metalloproteinase 9 (MMP9, ab76003, 1:1000, Abcam), anti-Vimentin (ab92547, 1:1000, Abcam), anti-E-cadherin (ab15148, 1:500, Abcam), anti-GAPDH (ab8245, 1: 500, Abcam), anti-Cleaved-caspase-3 (Cleaved-casp-3, ab2302, 1:1000, Abcam), anti- anti-Cleaved-caspase-9 (Cleaved-casp-9, ab2324, 1:2000, Abcam), anti-Multidrug Resistance associated Protein 1 (MRP1, ab32574, 1:500, Abcam), and anti-multidrug resistance-1 (MDR1, Ab170904, 1:1000, Abcam).

### Cell proliferation assay

The proliferation of PC-3/R and DU145/R cells was detected by 3-(4,5-dimethyl-2-thiazolyl)-2,5-diphenyl-2-H-tetrazolium bromide (MTT) assay. Briefly, transfected PC-3/R and DU145/R cells were seeded into 96-well plates and cultured for 24 h, 48 h, and 72 h. Then, MTT (20 μL, 5 mg/mL) was added to each wells and incubated for 4 h. Subsequently, dimethyl sulfoxide (200 μL) was utilized for the dissolution of the formazan crystals. In addition, PTX-resistant PCa cells were cultured in different PTX concentrations (0.2, 1, 5, 25, 125, 625, 1300 nM), and the half maximal inhibitory concentration (IC50) values of PTX-resistant PCa cells were assessed by MTT assay. Finally, the optical density (OD) was detected using a Microplate Reader (Thermo Fisher Scientific).

### Migration and invasion assays

The migration and invasion of transfected PC-3/R and DU145/R cells were determined using the transwell chamber (8 μm, Corning Costar, Corning, NY, USA). In short, transfected PC-3/R and DU145/R cells (1 × 10^6^) were individually seed into the RPMI 1640 medium in the upper chamber. The RPMI 1640 medium with FBS (10%) was added to the lower chamber as a chemoattractant. After 24 h cultivation, the migrated and invaded cells on the lower surface of the membrane were fixed via methanol (100%) and stained by crystal violet (0.1%). The invasion assay was executed using the transwell chamber coated with matrigel (BD Biosciences, San Jose, CA, USA). At last, the migrated or invaded cells were counted by the inverted microscope (Olympus, Tokyo, Japan).

### Cell apoptosis assay

The apoptosis of PC-3/R and DU145/R cells was assessed using an annexin V-fluorescein isothiocyanate (FITC) apoptosis-detection kit (Beyotime). Briefly, transfected PC-3/R and DU145/R cells were cultured with PTX (30 nM) for 48 h. After washing, the cells were re-suspended in binding solution with a concentration of 10^6^ cells/mL. Then, annexin V-FITC (10 μL) and propidiumiodide (5 μL) were added to the binding solution with PC-3/R and DU145/R cells and incubated for 20 min in the dark. In the end, the apoptosis rate of PC-3/R and DU145/R cells was determined using the FACScan^®^ flow cytometry (BD Biosciences).

### Dual-luciferase reporter assay

Bioinformatics database starBase v2.0 was applied to predict the binding sites of miR-186 in SNHG6. The pGL3-control vector (Promega, Madison, WI, USA) with the sequences of wild type SNHG6 (WT-SNHG6) (with predicted miR-186 binding sites) and mutant SNHG6 (MUT-SNHG6) were established to check on the binding sites between SNHG6 and miR-186. Then, the luciferase reporter vectors were cotransfected into PC-3/R and DU145/R with miR-NC or miR-186 using lipofectamine 2000 transfection reagent, respectively. At last, the luciferase activity of the luciferase reporter vectors was determined with luciferase reporter assay kit (Promega).

### RNA immunoprecipitation (RIP) assay

The Magna RIP kit (Millipore) was applied to verify the relationship between SNHG6 and miR-186. In short, RIP lysis buffer containing protease-inhibitor cocktail (Hoffman-La Roche, Basel, Switzerland) and RNase inhibitor (Thermo Fisher Scientific) was used for PC-3/R and DU145/R cell lysis. Then, the lysate of the PC-3/R and DU145/R cells was incubated with the RIP buffer harboring protein A/G magnetic beads conjugated IgG or Ago2 antibodies (Millipore) and incubated at 4 °C overnight. The enrichment of SNHG6 and miR-186 in precipitates was assessed with qRT-PCR.

### Tumor xenograft experiments

15 male BALB/c nude mice (4–6 weeks old) were obtained from Shanghai SLAC Laboratory Animal, co., Ltd. (Shanghai, China). Briefly, DU145/R cells carrying lentivirus-mediated sh-RNA targeting SNHG6 (sh-SNHG6) or negative control (sh-NC) (2 × 10^6^/0.2 mL PBS) were subcutaneously injected into the dorsal side of nude mice (5 mice/group) to establish the xenograft tumor models. After 7 days of injection, 5 mice carrying sh-SNHG6 were intraperitoneally injected with PTX (20 mg/kg, Sigma, Louis, Missouri, USA), once every 4 days, 5 times in total. Another 10 mice carrying sh-NC were intraperitoneally injected with PTX or phosphate buffered saline, which were used as control groups. After intraperitoneal injection, the tumor volume was measured every 4 days with a digital caliper and calculated with the equation: Volume = (length × width^2^)/2. After 27 days, the mice were euthanized for subsequent experiments. The procedures of xenograft assay were approved by the Ethics Committee of the Second People’s Hospital of Taizhou.

### Statistical analysis

SPSS 18.0 software (SPSS, Chicago, IL, USA) was utilized for statistical analysis. Pearson’s correlation analysis method was used for the analysis of the correlation between SNHG6 and miR-186. Data were exhibited as mean ± standard deviation. Differences with *P* < 0.05 were statistically significant. The differences between 2 or more groups were determined by Student’s *t* test or one-way variance analysis (ANOVA).

## Results

### SNHG6 was elevated while miR-186 was reduced in drug-resistant PCa tissues and cells

**Fig. 1:**
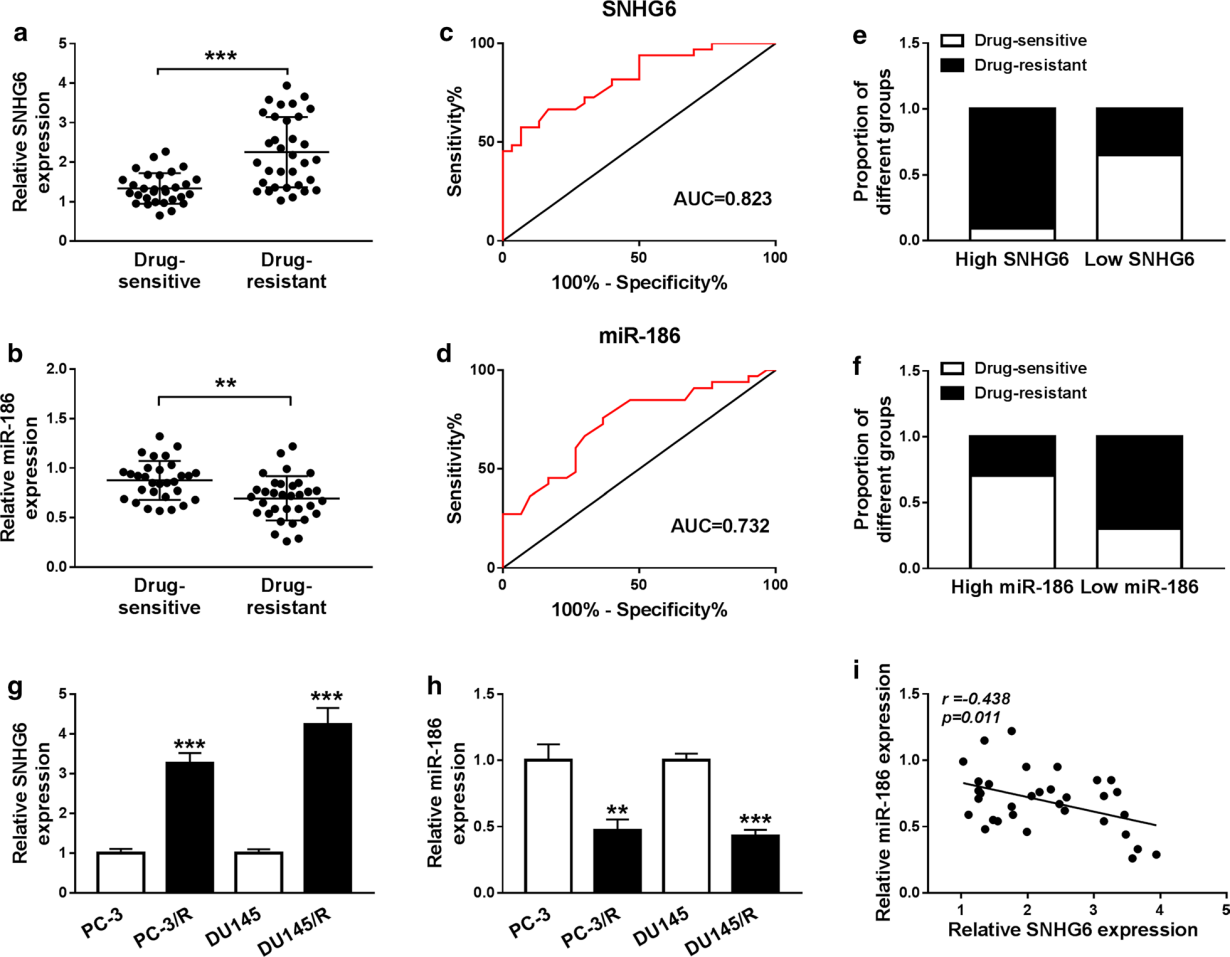
Levels of SNHG6 and miR-186 in drug-resistant PCa tissues and cells. **a**, **b** The expression of SNHG6 and miR-186 in 30 drug-sensitivity PCa tissues and 33 drug-resistant PCa tissues was detected using qRT-PCR. **c**, **d** ROC curve analysis exhibited the diagnostic value of SNHG6 and miR-186 for distinguishing drug-resistant patients from drug-sensitive patients. **e**, **f** The ratio of PTX-resistant PCa patients to PTX-sensitive PCa patients in high or low SNHG6 expression groups and low or high miR-186 groups based on the cut-off value of ROC. **g**, **h** The expression of SNHG6 and miR-186 in PC-3, PC-3/R, DU145, and DU145/R cells was evaluated by qRT-PCR. **i** Pearson’s correlation analysis was exhibited the correlation between SNHG6 and miR-186. **P < 0.001 and ***P < 0.001

In order to explore the relevance of SNHG6 in the chemo-resistance of PCa, we determined the expression pattern of SNHG6 and miR-186 in 30 drug-sensitivity PCa tissues and 33 drug-resistant PCa tissues by qRT-PCR. Compared with the drug-sensitivity tissues, the expression of SNHG6 was remarkably increased while miR-186 was distinctly decreased in drug-resistant PCa tissues (Fig. 1a, b). Furthermore, we utilized the receiver operating characteristic (ROC) curve to assess the diagnostic value of SNHG6 and miR-186 in serum. The ROC analysis revealed that the area under curve (AUC) of SNHG6 and miR-186 was 0.823 (95% confidence interval = 0.7227–0.9227 and the cutoff value was 1.935) and 0.732 (95% confidence interval = 0.6082–0.8564 and the cutoff value was 0.83), showing that SNHG6 and miR-186 might be diagnostic biomarkers for distinguishing drug-resistant patients from drug-sensitive patients (Fig. 1c, d). Additionally, the proportion of PCa patients with resistant to PTX was drastically higher in the high expression of SNHG6 group than that in the low expression of SNHG6 group according to the cutoff value of ROC (Fig. 1e). Inversely, the proportion of PCa patients with resistant to PTX was strikingly lower in the high miR-186 expression group compared to that in the low miR-186 expression group (Fig. 1f). We also assessed the expression of SNHG6 and miR-186 in PTX-resistant PCa cells by qRT-PCR. The results presented that SNHG6 was greatly increased while miR-186 was specially reduced in PC-3/R and DU145/R cells compared with the PC-3 and DU145 cells (Fig. 1g, h). Also, Pearson’s correlation analysis exhibited that the expression of SNHG6 and miR-186 in PCa tissues had a negative correlation (Fig. 1i). Therefore, we concluded that the negative correlation between SNHG6 and miR-186 in PCa tissues might be associated with PCa resistance, and SNHG6 and miR-186 served as potential diagnostic biomarkers for PCa.

### SNHG6 knockdown inhibited proliferation, migration, and invasion of PTX-resistant PCa cells

**Fig. 2:**
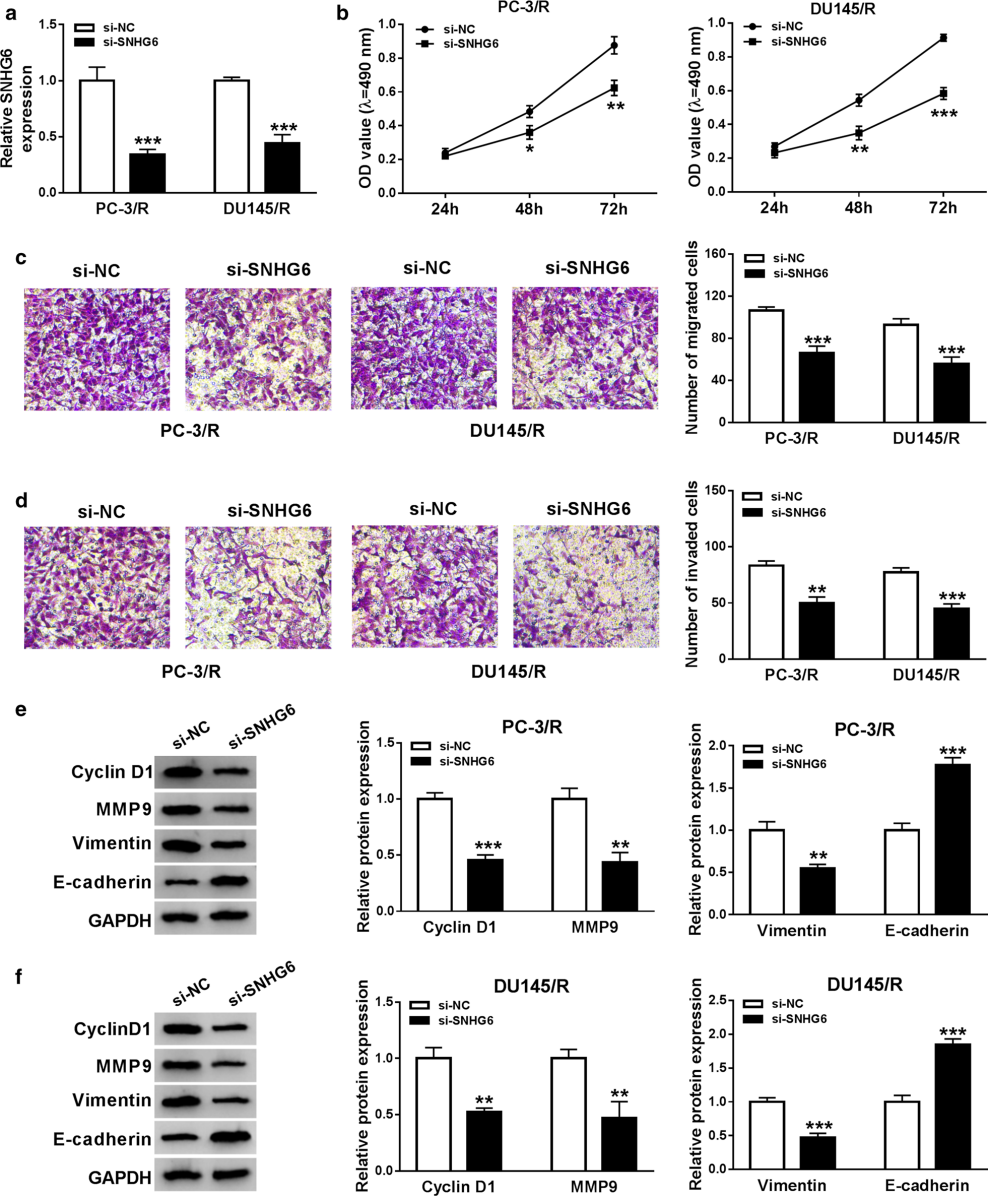
Impact of SNHG6 silencing on the proliferation, migration, and invasion of PTX-resistant PCa cells. **a**–**f** PC-3/R and DU145/R cells were transfected with si-SNHG6 or si-NC. **a** QRT-PCR was performed for the detection of the expression of SNHG6 in PC-3/R and DU145/R cells. **b** The proliferation ability of PC-3/R and DU145/R cells was assessed using MTT assay. **c**, **d** The migration and invasion capacities of PC-3/R and DU145/R cells were determined with transwell assay. **e**, **f** The levels of CyclinD1, MMP9, Vimentin, and E-cadherin in PC-3/R and DU145/R cells were detected with western blot analysis. **P < 0.001 and ***P < 0.001

Based on the above results, we performed loss-of-function to explore the role of SNHG6 in PTX-resistant PCa cells. First, PC-3/R and DU145/R cells were transfected with si-SNHG6 or si-NC to silence the expression of SNHG6. Results from qRT-PCR showed that SNHG6 expression was evidently reduced after transfection with si-SNHG6 in PC-3/R and DU145/R cells (Fig. 2a). Then, we further assessed the effects of SNHG6 inhibition on cell proliferation, migration, and invasion in PC-3/R and DU145/R cells. MTT assay indicated that the knockdown of SNHG6 conspicuously suppressed the proliferation of PC-3/R and DU145/R cells compared with the si-NC group (Fig. 2b). Furthermore, transwell assay displayed that the migration and invasion capacities of PC-3/R and DU145/R cells were dramatically repressed by the downregulation of SNHG6 compared to the control group (Fig. 2c, d). Subsequently, we detected the levels of CyclinD1, MMP9, Vimentin, and E-cadherin in SNHG6-sileced PC-3/R and DU145/R cells using western blot. As shown in Fig. 2e, f, silenced SNHG6 expression exceptionally decreased the levels of CyclinD1, MMP9, Vimentin and elevated the level of E-cadherin in PC-3/R and DU145/R cells. Additionally, the silencing of SNHG6 reduced the levels of ZEB1 and Snail in PC-3/R and DU145/R cells (Additional file 1: Fig. S1). In a summary, SNHG6 silencing could impede the proliferation, migration, and invasion of PTX-resistant PCa cells.

### SNHG6 silencing enhanced the sensitivity of PTX-resistant PCa cells to PTX

**Fig. 3:**
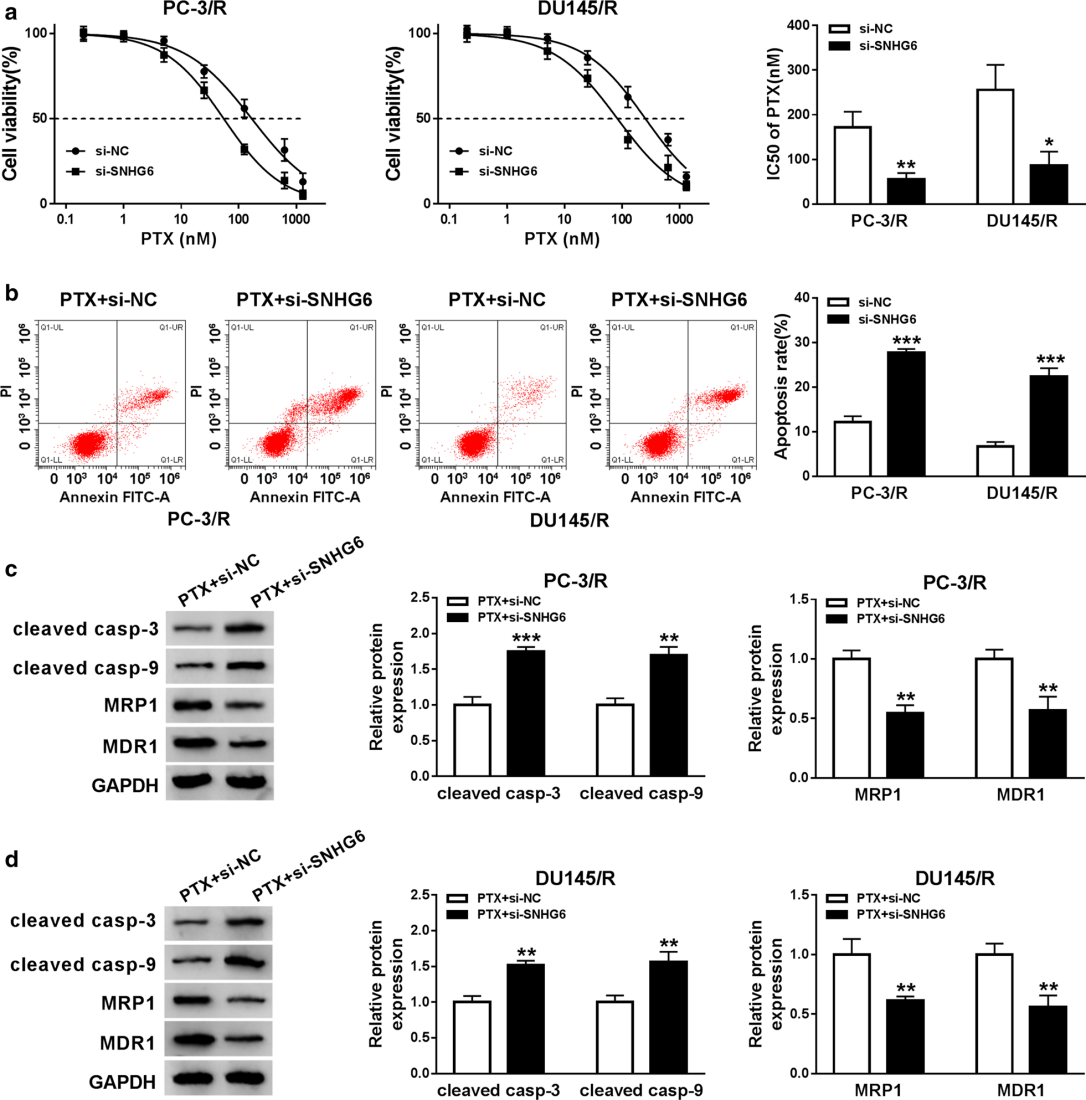
Effects of SNHG6 inhibition on the sensitivity of PTX-resistant PCa cells to PTX. **a**–**d** The si-SNHG6 or si-NC was transfected into PC-3/R and DU145/R cells. **a** The IC50 value of PC-3/R and DU145/R cells was assessed by MTT assay. **b** Flow cytometry assay was conducted to assess the apoptosis of PC-3/R and DU145/R cells with PTX (30 nM) treatment. **c**, **d** Western blot analysis was employed to measure the protein levels of cleaved casp-3, cleaved casp-9, MRP1, and MDR1 in PC-3/R and DU145/R cells with PTX treatment. *P < 0.05, **P < 0.001 and ***P < 0.001

Subsequently, we further explored the effect of SNHG6 inhibition on the sensitivity of PTX-resistant PCa cells to PTX. Firstly, PC-3/R and DU145/R cells transfected with si-NC or si-SNHG6 were cultured in medium with different PTX concentrations for 48 h. MTT assay presented that SNHG6 suppression obviously reduced IC_50_ value of PC-3/R and DU145/R in contrast to the si-NC group (Fig. 3a). We observed that SNHG6 inhibition specially contributed to the apoptosis of PC-3/R and DU145/R cells under PTX treatment compared to the control group (Fig. 3b). As we expected, impeded SNHG6 expression distinctly decreased the levels of MRP1, MDR1 and increased the levels of cleaved casp-3, cleaved casp-9 in PC-3/R and DU145/R cells under PTX treatment (Fig. 3c, d). These data indicated that SNHG6 downregulation could reinforce PTX-resistant PCa cells sensitivity to PTX.

### SNHG6 served as a sponge for miR-186 in PTX-resistant PCa cells

**Fig. 4:**
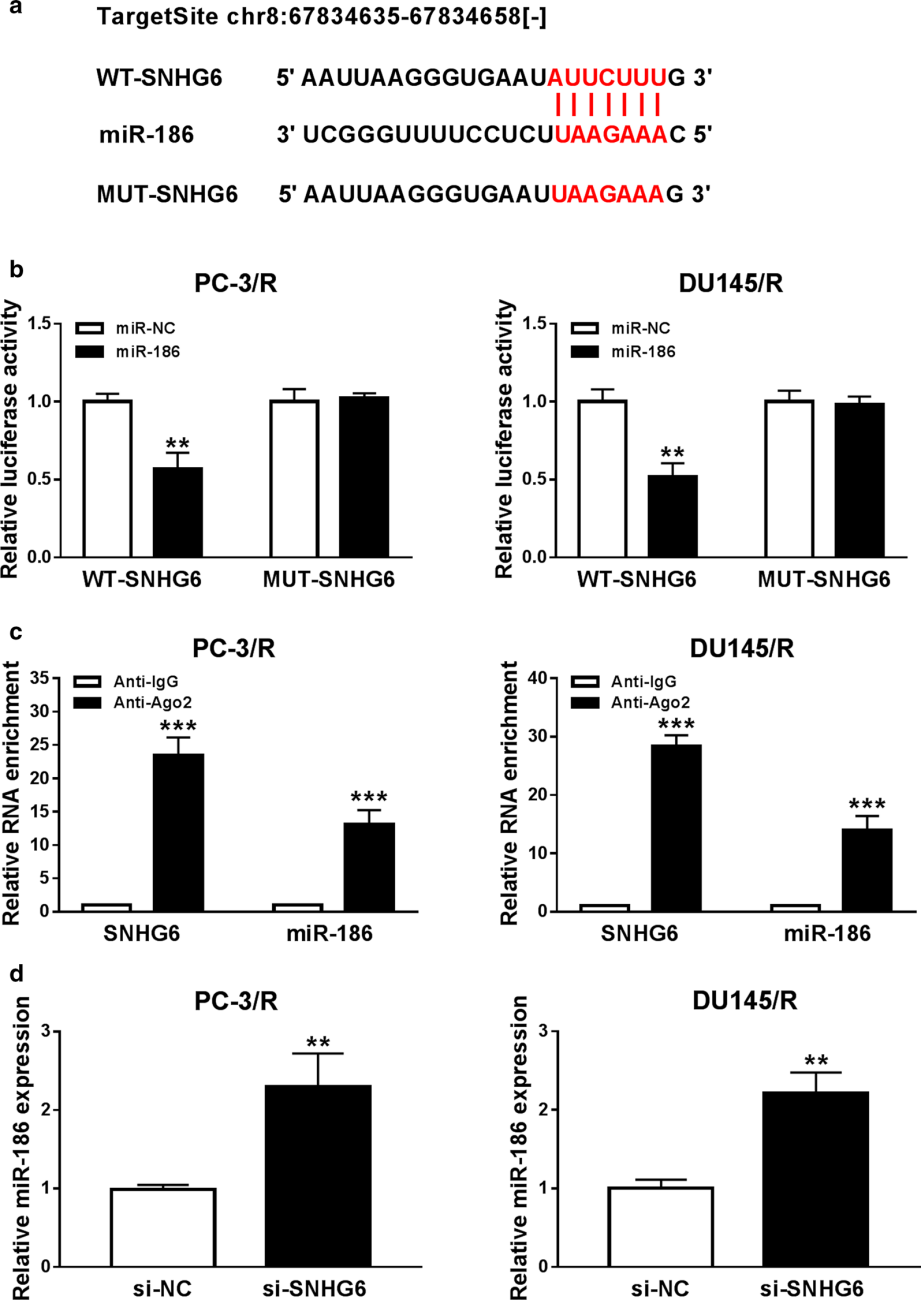
SNHG6 targeted miR-186 in PTX-resistant PCa cells. **a** Bioinformatics database starBase v2.0 was utilized to predict the binding sites between SNHG6 and miR-186. **b** Dual-luciferase reporter assay was performed to determine the luciferase activities of WT-SNHG6 and MUT-SNHG6 in PC-3/R and DU145/R cells transfected with miR-186 or miR-NC. **c** RIP was employed to verify the relationship between SNHG6 and miR-186 in PC-3/R and DU145/R cells. **d** QRT-PCR was applied to assess the expression of miR-186 in PC-3/R and DU145/R cells transfected with si-SNHG6 or si-NC. **P < 0.001 and ***P < 0.001

To survey the molecular mechanism of SNHG6 in PCa resistance, we predicted the potential targets of SNHG6 by the starBase v2.0 database. We discovered that miR-186 harbored complementary binding sites to SNHG6 (Fig. 4a). Dual-luciferase reporter assay revealed that the luciferase activity of WT-SNHG6 was markedly reduced in PC-3/R and DU145/R cells transfected with miR-186 compared to the miR-NC group. However, the luciferase activity of MUT-SNHG6 was no overt difference (Fig. 4b). RIP assay manifested that SNHG6 and miR-186 were gathered in Ago2-containing miRNA ribonucleoprotein complexes in contrast to IgG immunoprecipitates (Fig. 4c). Furthermore, SNHG6 silencing dramatically elevated the level of miR-186 in PC-3/R and DU145/R cells in comparison with the control group (Fig. 4d). Together, these results suggested that SNHG6 negatively regulated miR-186 expression in PTX-resistant PCa cells.

### MiR-186 inhibition reversed SNHG6 silencing-mediated proliferation, migration, and invasion of PTX-resistant PCa cells

**Fig. 5:**
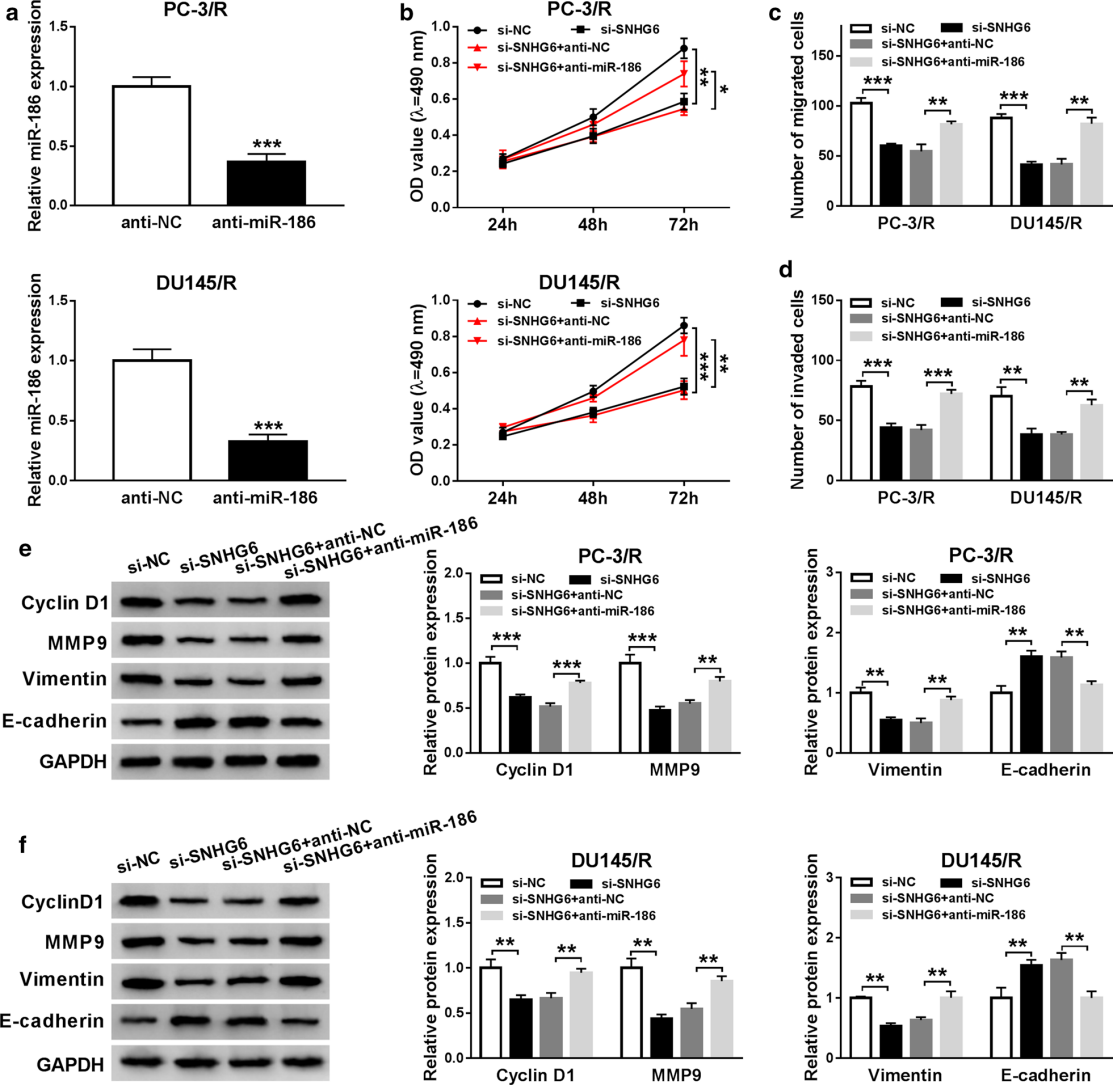
SNHG6 mediated the proliferation, migration, and invasion of PTX-resistant PCa cells through miR-186. **a** QRT-PCR was utilized for the assessment of miR-186 expression in PC-3/R and DU145/R cells transfected with anti-NC or anti-miR-186. **b**–**f** PC-3/R and DU145/R cells were transfected with si-NC, si-SNHG6, si-SNHG6 + anti-NC, or si-SNHG6 + anti-miR-186. **b** MTT assay was conducted for the detection of the proliferation of PC-3/R and DU145/R cells. **c**, **d** Transwell assay was executed to determine the migration and invasion of PC-3/R and DU145/R cells. **e**, **f** western blot analysis was used to detect the levels of CyclinD1, MMP9, Vimentin, and E-cadherin in PC-3/R and DU145/R cells. *P < 0.05, **P < 0.001 and ***P < 0.001

To determine whether SNHG6 impacted cell proliferation, migration, and invasion via miR-186 in PTX-resistant PCa cells, we employed qRT-PCR to analyze the expression of miR-186 in PC-3/R and DU145/R cells transfected with anti-NC or anti-miR-186. The results demonstrated that miR-186 expression was downregulated in PC-3/R and DU145/R cells transfected with anti-miR-186 (Fig. 5a). MTT assay exhibited that the inhibition of miR-186 overturned the inhibitive effect of SNHG6 silencing on cell proliferation in PC-3/R and DU145/R cells (Fig. 5b). Transwell assay revealed that miR-186 inhibitor overturned the repression of migration and invasion of PC-3/R and DU145/R cells following the downregulation of SNHG6 (Fig. 5c, d). Also, western blot analysis disclosed that both the reduction of CyclinD1, MMP9, Vimentin and the elevation of E-cadherin in PC-3/R and DU145/R cells were reversed by the knockdown of miR-186 (Fig. 5e, f). In addition, silenced SNHG6 expression decreased the levels of ZEB1 protein in PC-3/R and DU145/R cells, while this influence was reversed after miR-186 silencing (Additional file 2: Fig. S2). These findings manifested that SNHG6 impacted the proliferation, migration, and invasion of PTX-resistant PCa cells via miR-186.

### MiR-186 inhibitor restored SNHG6 silencing-mediated the sensitivity of PTX-resistant PCa cells to PTX

**Fig. 6:**
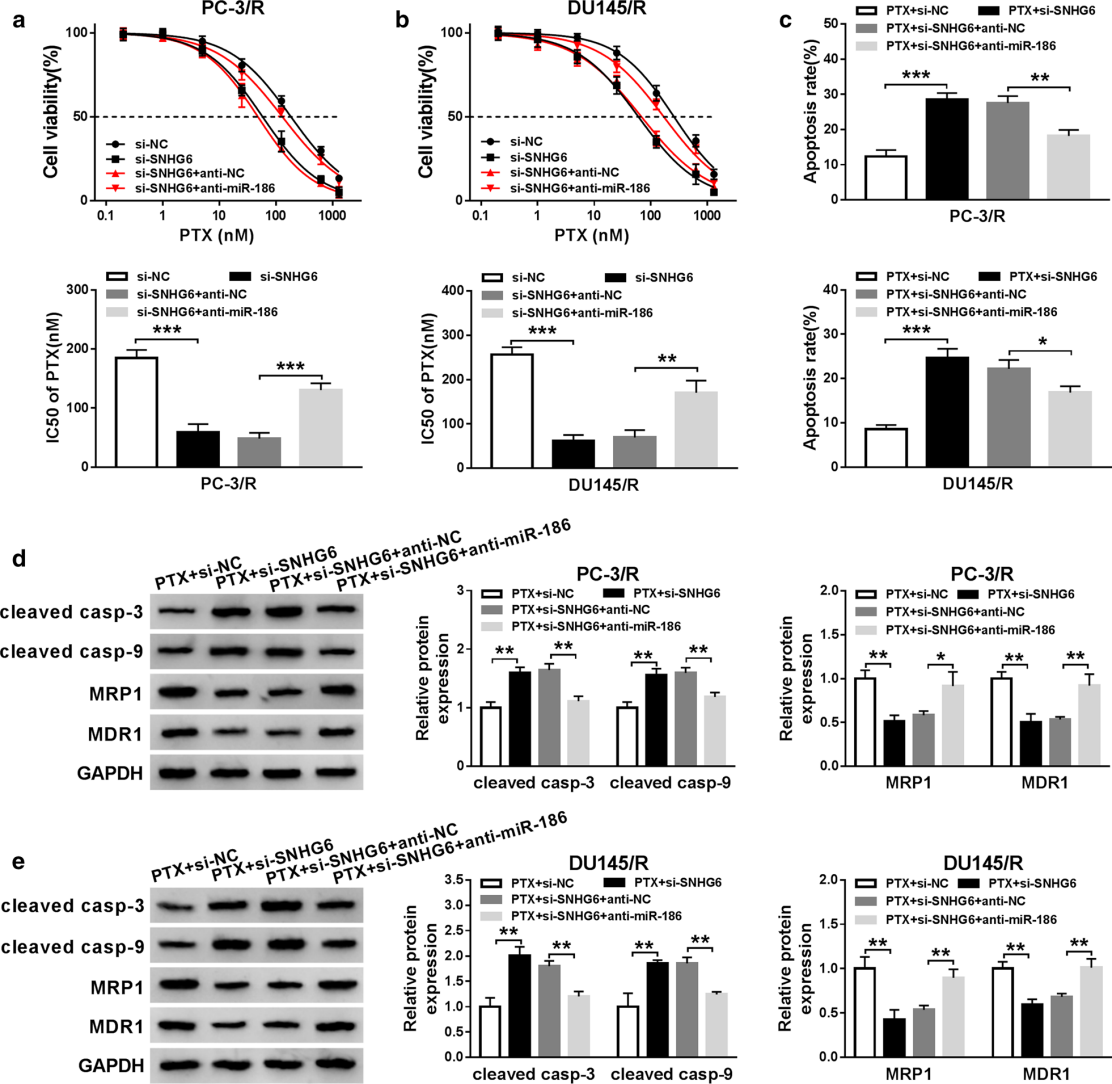
SNHG6 regulated the sensitivity of PTX-resistant PCa cells to PTX by miR-186. **a**–**e** The si-NC, si-SNHG6, si-SNHG6 + anti-NC, or si-SNHG6 + anti-miR-186 was transfected into PC-3/R and DU145/R cells. **a**, **b** MTT assay was used to analyze the IC50 value of PC-3/R and DU145/R cells. **c** The apoptotic rate of PC-3/R and DU145/R under PTX (30 nM) treatment was detected through flow cytometry assay. **d**, **e** The levels of cleaved casp-3, cleaved casp-9, MRP1, and MDR1 in PC-3/R and DU145/R cells under PTX (30 nM) treatment was evaluated with western blot analysis. *P < 0.05, **P < 0.001 and ***P < 0.001

Based on the above results, we explored whether SNHG6 impacted the sensitivity of PTX-resistant PCa cells to PTX through miR-186. At the outset, the IC_50_ value of PC-3/R and DU145/R cells transfected with si-NC, si-SNHG6, si-SNHG6 + anti-NC, or si-SNHG6 + anti-miR-186 was analyzed through MTT assay under PTX treatment. The results presented that the decrease of IC_50_ value of PC-3/R and DU145/R cells caused by SNHG6 inhibition was reverted by the inhibition of miR-186 expression (Fig. 6a, b). Subsequently, flow cytometry assay manifested that miR-186 inhibition overturned the acceleration of apoptosis of PC-3/R and DU145/R cells induced by SNHG6 knockdown under PTX treatment (Fig. 6c). Besides, western blot analysis revealed that the effects of SNHG6 silencing on the levels of cleaved casp-3, cleaved casp-9, MRP1, and MDR1 of PC-3/R and DU145/R cells under PTX treatment were reversed by the repression of miR-186 expression (Fig. 6d, e). Therefore, these data suggested that SNHG6 modulated the sensitivity of PTX-resistant PCa cells to PTX by miR-186.

### Knockdown of SNHG6 elevated PCa sensitivity to PTX in vivo

**Fig. 7:**
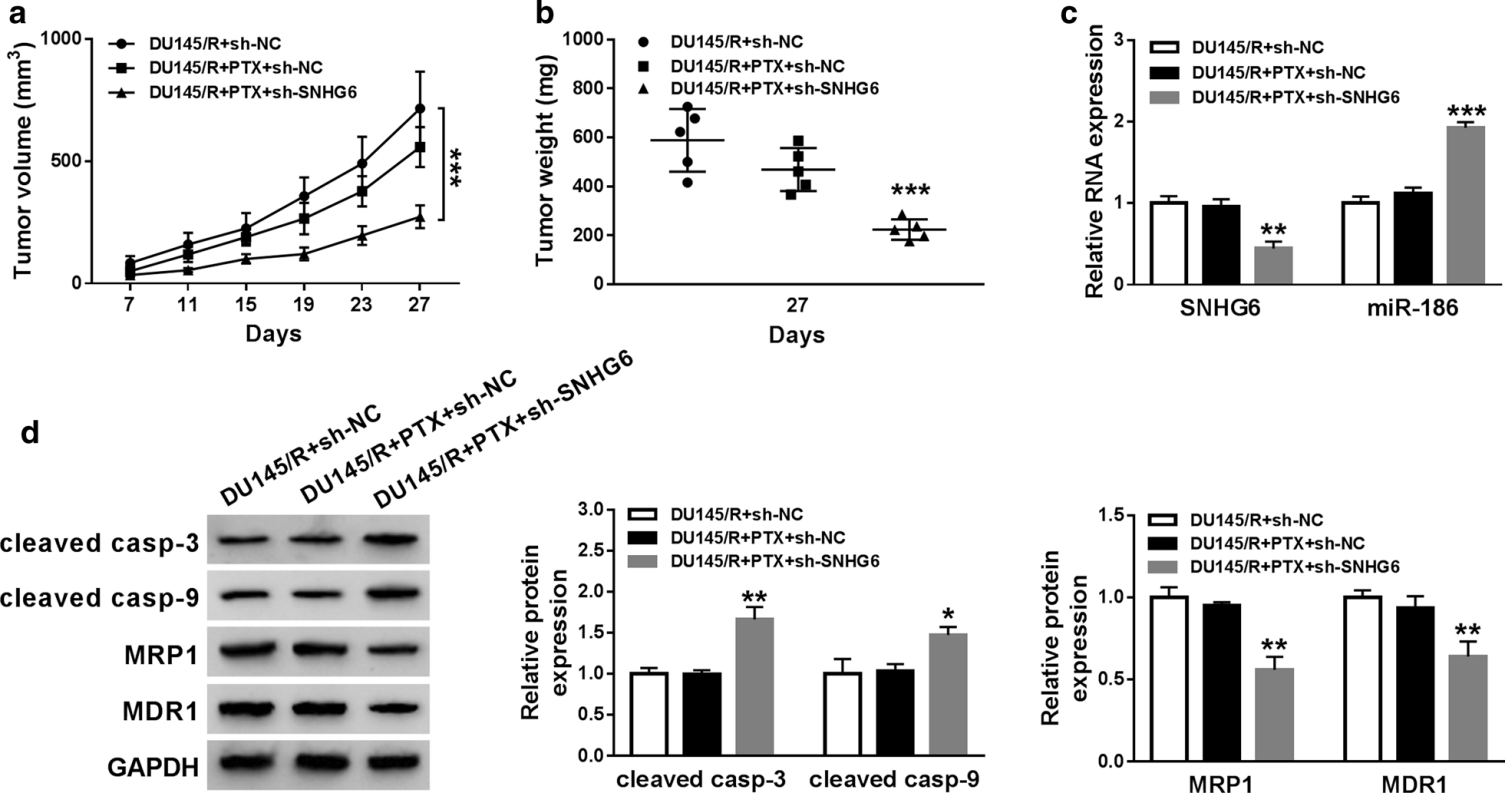
Knockdown of SNHG6 elevated the sensitivity of PTX-resistant PCa cells to PTX in vivo. **a** The curves of tumor volume in the sh-SNHG6 and sh-NC groups with or without PTX treatment. **b** Mice tumor weight in the sh-SNHG6 and sh-NC groups with or without PTX treatment. **c** The expression levels of SNHG6 and miR-186 in mice tumor tissues of the sh-SNHG6 and sh-NC groups with or without PTX treatment were analyzed by qRT-PCR. **d** Western blot analysis of the levels of cleaved casp-3, cleaved casp-9, MRP1, and MDR1 in mice tumor tissues of the sh-SNHG6 and sh-NC groups with or without PTX treatment. *P < 0.05, **P < 0.001 and ***P < 0.001

To confirm the role of SNHG6 on the sensitivity of PTX-resistant PCa cells to PTX in vivo, the xenograft model of DU145/R cells was established. The nude mice were subcutaneously injected with DU145/R cells carrying sh-SNHG6 or sh-NC and then treated with PTX. The results demonstrated that PTX treatment effectively impeded tumor growth in the sh-SNHG6 group compared with the sh-NC group with or without PTX treatment (Fig. 7a, b). Then, we further disclosed that SNHG6 was downregulated and miR-186 was upregulated in the sh-SNHG6 group with PTX treatment compared to the control groups (Fig. 7c). In addition, the levels of cleaved casp-3 and cleaved casp-9 were elevated and the levels of MRP1 and MDR1 were decreased in mice tumor tissues of the sh-SNHG6 group with PTX treatment in comparison to the control groups (Fig. 7d). Taken together, these results revealed that SNHG6 inhibition elevated the sensitivity of PTX-resistant PCa cells to PTX in vivo.

## Discussion

PCa is the second most common tumor among males, which seriously threatens men’s health and life. PTX can destroy microtubules and inhibit cell division, but its severve side effects can rapidly develop PCa patients resistant to drugs [25, 26]. Consequently, it is necessary to develop new targeted therapies to combat PCa resistance.

LncRNAs have been reported as promising tumor biomarkers or therapeutic targets [27]. In the past few years, a series of evidence has revealed that lncRNAs exert crucial roles in the chemo-resistance of multiple malignant tumors, including PCa [28]. SNHG6 was pointed out to play a carcinogenic role in diverse cancers. For instance, SNHG6 facilitated cancer cell migration and proliferation in ovarian clear cell cancer [29], breast cancer [13], and lung adenocarcinoma [30]. Also, report of Wang et al. disclosed SNHG6 increased the resistance of colorectal cancer to 5-fluorouracial and repressed 5-fluorouracial-induced apoptosis and expedited autophagy [15]. In the current study, SNHG6 expression was elevated in drug-resistant PCa tissues and cells and might be a diagnostic biomarker for distinguishing drug-resistant patients from drug-sensitive patients in PCa. Moreover, SNHG6 silencing blocked the proliferation, migration, and invasion of PTX-resistant PCa cells in vitro, and enhanced the sensitivity of PTX-resistant PCa cells to PTX in vitro and in vivo. These data indicated that SNHG6 increased PCa chemo-resistance to PTX.

Studies have shown that lncRNAs are involved in the chemo-resistance of cancers by acting as sponge for miRNAs [7, 31, 32]. Herein, we discovered and confirmed that miR-186 was a target of SNHG6 by starBase v2.0, dual-luciferase reporter assay, and RIP assay. One study showed that miR-186 was decreased in drug-resistant non-small cell lung cancer tissues, and elevated miR-186 expression increased the sensitivity of non-small cell lung cancer cells to PTX [23]. Zhu et al. reported that the overexpression of miR-186 increased the sensitivity of ovarian cancer cell to cisplatin in vitro and in vivo and inhibited cell mesenchymal-to-epithelia, cell cycle progression, and promoted cell apoptosis [22]. In this study, miR-186 was reduced in drug-resistant PCa tissues and cells. Also, miR-186 was a latent diagnostic biomarker for distinguishing drug-resistant patients from drug-sensitive patients in PCa. Furthermore, inhibited miR-186 expression reversed SNHG6 knockdown-mediated effects on cell proliferation, migration, invasion, and PTX sensitivity in PTX-resistant PCa cells. These data proved that SNHG6 regulated the resistance of PTX-resistant PCa cells via sponging miR-186.

## Conclusion

SNHG6 was upregulated while miR-186 was downregulated in resistant PCa tissues and cells. SNHG6 sponged miR-186 to reduce cell PTX sensitivity, elevated proliferation, migration, and invasion in PTX-resistant PCa cells. Therefore, our research provided evidence for SNHG6 as a latent target for PCa treatment.

## Supplementary information

**Additional file 1: Fig. S1.** Influence of SNHG6 knockdown on the levels of ZEB1 and Snail in PTX-resistant PCa cells. The levels of in ZEB1 and Snail in PC-3/R and DU145/R cells transfected with si-NC or si-SNHG6 were evaluated by western blot.

**Additional file 2: Fig. S2.** SNHG6 regulated ZEB1 expression via miR-186 in PTX-resistant PCa cells. The level of ZEB1 protein in PC-3/R and DU145/R cells transfected with si-NC, si-SNHG6, si-SNHG6 + anti-NC or si-SNHG6 + anti-miR-186 was evaluated by western blot.

## Data Availability

The datasets used and/or analyzed during the current study are available from the corresponding author on reasonable request.
