# Supplementary figures and images for: Long non-coding RNA SNHG6 regulates the sensitivity of prostate cancer cells to paclitaxel by sponging miR-186
Source: Cancer Cell Int. 2020 Aug 7;20:381. doi: 10.1186/s12935-020-01462-x (PMC7412850; doi:10.1186/s12935-020-01462-x)

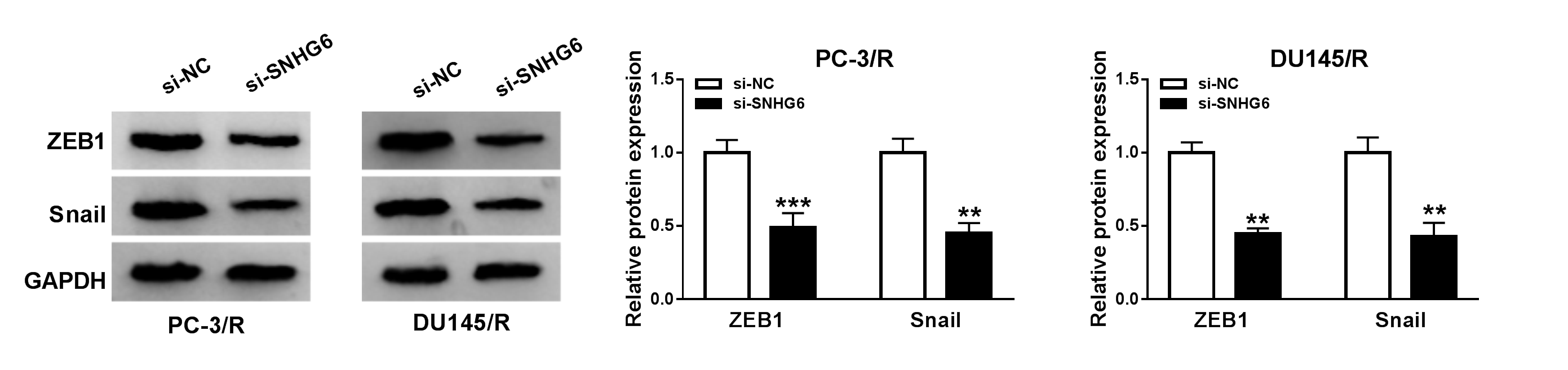

Supplement: Supplementary file 1 — Additional file 1: Fig. S1. Influence of SNHG6 knockdown on the levels of ZEB1 and Snail in PTX-resistant PCa cells. The levels of in ZEB1 and Snail in PC-3/R and DU145/R cells transfected with si-NC or si-SNHG6 were evaluated by western blot. [file 12935_2020_1462_MOESM1_ESM.tif]

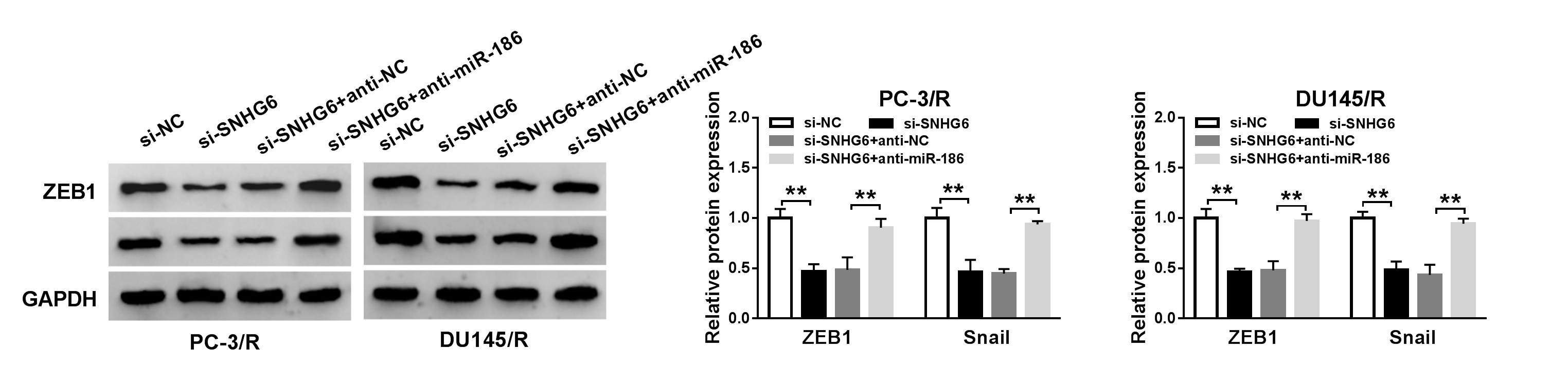

Supplement: Supplementary file 2 — Additional file 2: Fig. S2. SNHG6 regulated ZEB1 expression via miR-186 in PTX-resistant PCa cells. The level of ZEB1 protein in PC-3/R and DU145/R cells transfected with si-NC, si-SNHG6, si-SNHG6 + anti-NC or si-SNHG6 + anti-miR-186 was evaluated by western blot. [file 12935_2020_1462_MOESM2_ESM.tif]
